# Evaluation of a Novel Liquid Fiducial Marker, BioXmark^®^, for Small Animal Image-Guided Radiotherapy Applications

**DOI:** 10.3390/cancers12051276

**Published:** 2020-05-18

**Authors:** Kathryn H. Brown, Mihaela Ghita, Giuseppe Schettino, Kevin M. Prise, Karl T. Butterworth

**Affiliations:** 1 Patrick G. Johnston Centre for Cancer Research, Queen’s University Belfast, Belfast BT9 7AE, UK; m.ghita@qub.ac.uk (M.G.); k.prise@qub.ac.uk (K.M.P.); k.butterworth@qub.ac.uk (K.T.B.); 2 Department of Physics, Faculty of Engineering and Physical Sciences, University of Surrey, Guildford, Surrey GU2 7XH, UK; giuseppe.schettino@surrey.ac.uk; 3 National Physical Laboratory, Hampton Road, Teddington, Middlesex TW11 0LW, UK

**Keywords:** small animal image-guided radiotherapy, fiducial markers, radiation response

## Abstract

BioXmark^®^ (Nanovi A/S, Denmark) is a novel fiducial marker based on a liquid, iodine-based and non-metallic formulation. BioXmark^®^ has been clinically validated and reverse translated to preclinical models to improve cone-beam CT (CBCT) target delineation in small animal image-guided radiotherapy (SAIGRT). However, in phantom image analysis and in vivo evaluation of radiobiological response after the injection of BioXmark^®^ are yet to be reported. In phantom measurements were performed to compare CBCT imaging artefacts with solid fiducials and determine optimum imaging parameters for BioXmark^®^. In vivo stability of BioXmark^®^ was assessed over a 5-month period, and the impact of BioXmark^®^ on in vivo tumour response from single-fraction and fractionated X-ray exposures was investigated in a subcutaneous syngeneic tumour model. BioXmark^®^ was stable, well tolerated and detectable on CBCT at volumes ≤10 µL. Our data showed imaging artefacts reduced by up to 84% and 89% compared to polymer and gold fiducial markers, respectively. BioXmark^®^ was shown to have no significant impact on tumour growth in control animals, but changes were observed in irradiated animals injected with BioXmark^®^ due to alterations in dose calculations induced by the sharp contrast enhancement. BioXmark^®^ is superior to solid fiducials with reduced imaging artefacts on CBCT. With minimal impact on the tumour growth delay, BioXmark^®^ can be implemented in SAIGRT to improve target delineation and reduce set-up errors.

## 1. Introduction

Radiotherapy is a major modality in the radical treatment of cancer, being prescribed to >50% of patients during their treatment [1]. In recent decades, significant advances in radiotherapy technology have enabled increasingly sophisticated, conformal delivery methods to be implemented into routine clinical practice, such as intensity modulated radiotherapy (IMRT), volumetric modulated arc therapy (VMAT) and stereotactic body radiotherapy (SBRT) [2,3,4]. The success of high-precision radiotherapy techniques is predicated on the use of volumetric imaging methods at all stages of the treatment process, from planning, delivery and verification to follow-up. Parallel developments in small animal image-guided radiotherapy (SAIGRT) platforms presents the opportunity to closely mimic clinical scenarios and further radiobiological understanding of tumour and normal tissue response [5]. Although these platforms increase the ability to irradiate small target volumes in vivo with the highest precision and accuracy to date, there is scope for improvement of soft tissue imaging and treatment alignment parameters.

Several imaging modalities have been used to optimise treatment positioning accuracy and precision, including megavoltage planar imaging, static kilovoltage planar imaging, ultrasound, cone beam CT (CBCT) and portal imaging [6,7,8]. However, these methods have limited soft-tissue contrast, which has driven the recent integration of magnetic resonance imaging (MRI) with linear accelerators (Linac) in the MR-Linac Radiotherapy Systems. These systems have the potential to exploit the benefits of MRI with real-time motion management and adjustment on a patient-specific basis [9]. The clinical benefits of MRI-guided radiotherapy remain to be fully demonstrated, and it is unlikely this approach will be widely implemented into routine practice for most tumours in the short term.

Currently, solid fiducial markers are FDA-approved and used in clinical practice to improve the visualization of low-contrast tissues and reduce uncertainties in set-up and targeting [10,11,12,13,14,15]. However, solid fiducial markers can negatively impact CT and MRI due to differences in image artefacts and contrast enhancement [16,17,18,19,20]. Furthermore, imaging artefacts may increase the dosimetric error during treatment planning and are a particular concern in proton therapy [21,22].

Recently, a novel liquid fiducial marker, BioXmark^®^, was developed and demonstrated to produce fewer imaging artefacts in multiple imaging modalities [16,18,22,23]. BioXmark^®^ was been applied to preclinical imaging studies in an orthotopic pancreatic tumour model and in phantom measurements [23,24,25]. There is further scope to increase precision and accuracy in SAIGRT studies and to improve animal welfare within the framework of the National Centre for the Replacement, Refinement and Reduction of Animals in Research (NC3Rs) [26,27,28].

The purpose of this study was to compare image contrast and artefacts of BioXmark^®^ with current solid fiducial markers used for preclinical CBCT imaging applications. We also aimed to determine the stability of BioXmark^®^ and evaluate its impact on tumour response in vivo.

## 2. Results

### 2.1. In Phantom Studies

**Figure 1:**
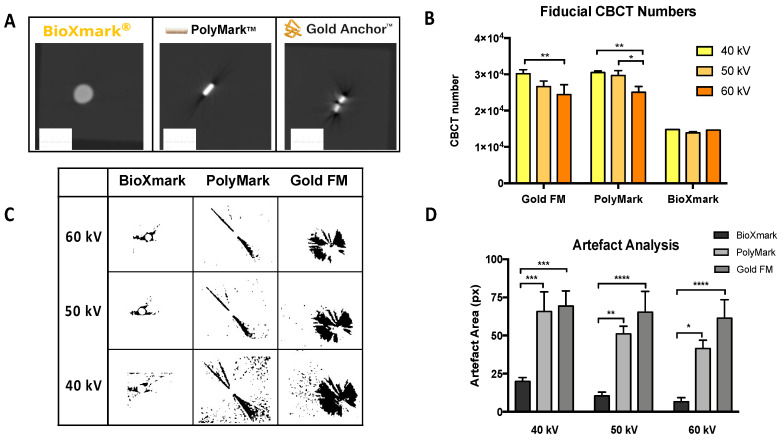
In phantom analysis of CBCT visibility and imaging artefacts for BioXmark^®^ and solid fiducial markers. (**A**) Representative imaging of BioXmark^®^ (20 μL), polymer and gold fiducial markers and corresponding CBCT numbers at imaging energies of 40, 50 and 60 kV (**B**). (**C**) Analysis of imaging artefacts on CBCT images processed using ImageJ and presented at imaging energies of 40, 50 and 60 kV (**D**). Data presented are mean values ± standard error (SEM); statistical significance is reported as * > 0.05, ** > 0.01, *** 0.0001, **** > 0.0001.

Imaging artefacts can be defined as discrepancies in CBCT scans that are not present in the object under investigation and degrade the quality of the image. These reduce the visualisation of structures through streaks, lines and shadows [29]. Imaging artefacts following CBCT were determined at energies of 40, 50 and 60 kV (0.5 mm Al filtration) for BioXmark^®^ and two clinically used solid fiducial markers (Figure 1A). Fiducials were placed in a 3D-printed box which was filled with gelatine to replicate soft tissue density. All three fiducials were easily visualised on CBCT at each of the energies investigated, which are typically used during small animal imaging (40–60 kV). CBCT numbers ranging between 12,000 and 33,000 were detected across all three of the markers investigated. CBCT numbers for the solid fiducials were 40%–53% (*p* < 0.001) higher than those detected for BioXmark^®^ (Figure 1B), which indicated a higher X-ray attenuation [30]. Imaging artefacts showed a distinct energy dependence, with the lowest imaging artefacts for all fiducial markers observed at 60 kV. Comparing energies of 40 kV and 60 kV, there were reductions of 12% (*p* = 0.61), 37% (*p* = 0.12) and 66% (*p* < 0.001) of imaging artefact area for gold, polymer and BioXmark^®^, respectively (Figure 1C,D). Overall, imaging artefacts across all energies caused by BioXmark^®^ were significantly lower than both of the solid markers, with a reduction of 89% (*p* < 0.001) and 84% (*p* < 0.001) imaging artefact area compared to gold and polymer fiducials at 60 kV. Although the solid fiducial markers had good contrast enhancement, the observed increase in imaging artefacts compared to BioXmark^®^ negatively impacted the imaging of anatomical structures, which is important during SAIGRT.

**Figure 2:**
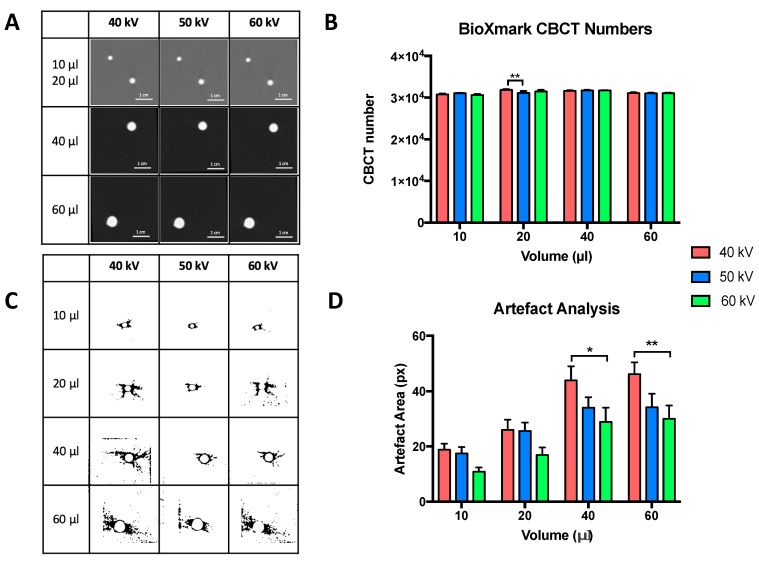
In phantom analysis of CBCT visibility and imaging artefacts for multiple volumes of BioXmark^®^. (**A**) Representative imaging of 10, 20, 40 and 60 μL of BioXmark^®^ on CBCT at 40, 50 and 60 kV. CBCT scans were taken twice at each energy. Corresponding CBCT numbers at imaging energies of 40, 50 and 60 kV (**B**). Analysis of imaging artefacts were processed on ImageJ (**C**) and quantified (**D**). Data presented are mean values ± SEM; statistical significance is reported as * > 0.05, ** > 0.01.

To further analyse CBCT imaging artefacts, multiple volumes of BioXmark^®^ were compared using the same in phantom set-up (Figure 2). Figure 2A shows the CBCT images for volumes of BioXmark^®^ from 10 to 60 μL, which are more suitable for SAIGRT. All volumes were easily visualised with minimal difference in CBCT values (Figure 2B); a minor fluctuation of 2.0% ± 2.4% was seen between 40 kV and 50 kV for 20 μL. Imaging artefacts were shown to positively correlate with the volume of BioXmark^®^ (Figure 2C,D). However, the observed imaging artefacts at the largest volume of 60 μL were substantially lower than those for the solid markers, which had a comparable volume of 0.4–2.4 µL. These data are consistent with observations from previous studies which concluded that volumes of BioXmark^®^ >50 μL produce hardening imaging artefacts in vivo, making it unsuitable for SAIGRT [24]. Our results suggest that volumes >60 μL produce imaging artefacts comparable to that of solid fiducial markers, whilst smaller volumes (10 and 20 μL) can effectively enhance CBCT image contrast enhancement by at least 50% with minimal surrounding imaging artefacts. These data support and define key parameters for the application of BioXmark^®^ in SAIGRT.

### 2.2. In Vivo Stability

**Figure 3:**
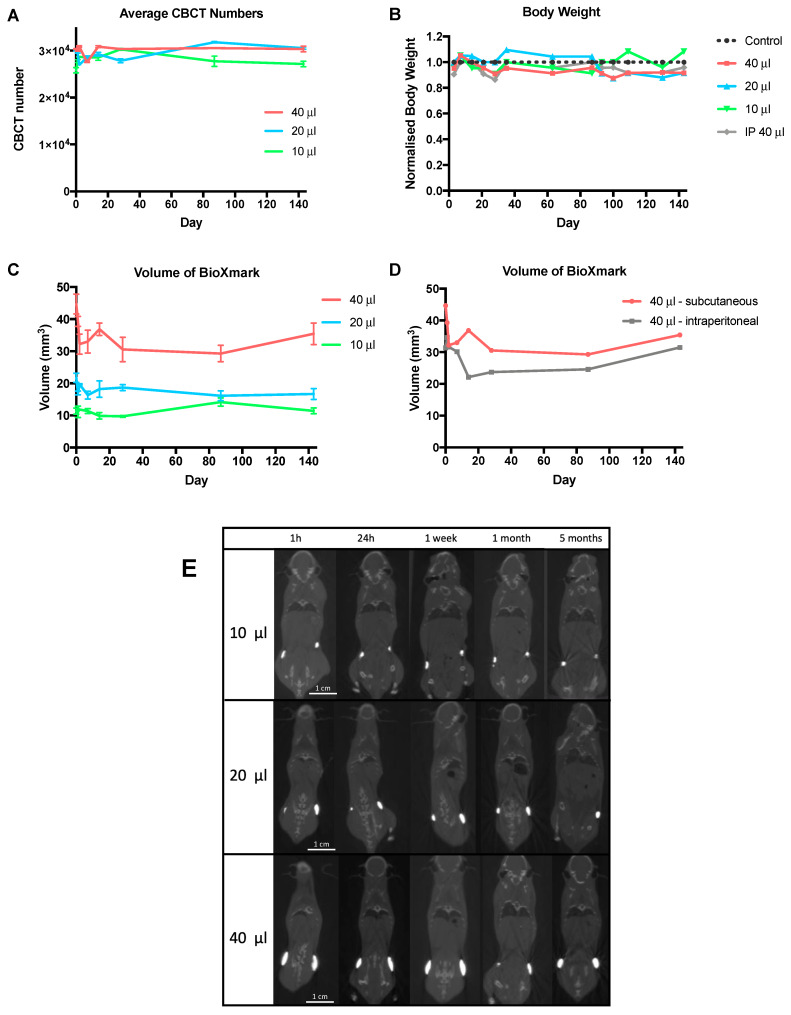
Evaluation of BioXmark^®^ in vivo over a 5-month period. (**A**) CBCT visibility of different volumes of BioXmark^®^ (10, 20 and 40 μL) subcutaneously injected were assessed over a 5-month period along with corresponding marker volumes (average ± SEM, *n* = 2) (**C**). (**D**) Comparison of marker volumes after a 40 μL subcutaneous or intraperitoneal injection. (**B**) Health and wellbeing of mice was monitored through weight measurements. No changes to marker shape and movement were detected via representative coronal CBCT scans of BioXmark^®^ injected subcutaneously (**E**).

We tested the long-term stability of BioXmark^®^ in vivo with longitudinal CBCT imaging analysis over a 5-month period following subcutaneous and intraperitoneal injection. Figure 3 shows the CBCT values, volume of BioXmark^®^ and body weights of mice throughout the duration of the study. Body weight, body score conditioning and the behaviour of mice was normal, with BioXmark^®^ not having an effect on the viability of the mice (Figure 3B). BioXmark^®^ was easily observable on CBCT and maintained a high CBCT value for each volume, with no significant marker degradation evident up to 5 months post-injection in either subcutaneous or intraperitoneal models (Figure 3A).

The observed volumes of BioXmark^®^ showed a small decrease after injection but then remained stable for the remainder of the study, with minimal tissue migration seen on CBCT scans (Figure 3C–E). This was expected due to the efflux of the solvent, ethanol, causing an immediate decrease in marker volume [31]. An average decrease in volume of 13% (*p* = 0.40), 16.1% (*p* = 0.35) and 17.5% (*p* = 0.17) was detected 14 days post-injection for 10 μL, 20 μL and 40 μL, respectively.

Volume changes were monitored after a subcutaneous or intraperitoneal injection of 40 μL (Figure 3D). Higher stability was apparent in the subcutaneous injection due to insignificant marker migration and constant volume. Although the volume of BioXmark^®^ decreased after intraperitoneal injection, the volume was stable with no decrease in marker volume for the remainder of the study. In agreement with previously published reports, 10 μL was found to be the smallest volume that could be reproducibly injected [24].

Migration of subcutaneously injected markers was visually assessed from CBCT scans and quantified from a defined reference points in the spine. All volumes were shown to be stable with all movements being less than 2 mm (Figure 3E).

Intraperitoneal injection led to movement of the marker throughout the peritoneal cavity; this may have been due to the marker accumulating in small volumes at different areas (Figure S1).

### 2.3. In Vivo Tumour Model

**Figure 4:**
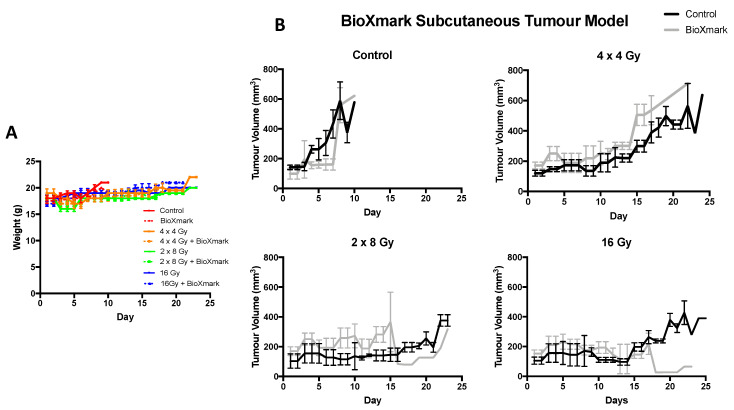
Impact of BioXmark^®^ on LLC tumour growth delay following single-fraction and fractioned radiotherapy exposures. Mice were intra-tumorally injected with 20 μL of BioXmark^®^ prior to irradiation. (**A**) Mean animal weight per treatment group versus time (days). (**B**) Data are presented as median tumour volume for the experimental group ± SEM versus time in days (average six mice per experimental group).

C57BL/6 mice subcutaneously implanted with Lewis lung carcinoma (LLC) tumours were monitored (weight and tumour volume) three times weekly (Figure 4). Mice weights within each treatment group showed minor fluctuations but remained within a tolerable weight loss of <15% (Figure 4A). At tumour volumes of 100 mm^3^, mice were randomly assigned to treatment groups as defined in the Materials and Methods. A previous preclinical study with BioXmark^®^ demonstrated a successful orthotopic injection of the marker after the tumour had grown to a treatable size [24]. Although, clinically, fiducial markers are usually placed in tissue surrounding a tumour, our study aimed to evaluate radiobiological response of BioXmark^®^ on tumour tissue; therefore, we completed intratumoral injections for beam targeting. BioXmark^®^ was not injected with the tumour cells as this was deemed unfeasible due to a high viscosity and semi-hydrophobic composition in a previous study [24]. Once the tumours were established at a pre-treatment volume of 100 mm^3^, BioXmark^®^ was injected intratumorally. We suspected no uptake of the marker into tumour cells, but a small volume of cells would be surrounded by BioXmark^®^. Treatment plans for subgroups without an intratumoral injection of BioXmark^®^ were targeted to the central point of the tumour.

Control animals had rapidly growing proliferative tumours which were not significantly impacted by intratumoral injection of BioXmark^®^ and reached maximum tumour volume within 10 days (*p* = 0.55). After a single fraction of 16 Gy, both control and BioXmark^®^-injected animals showed an average tumour growth delay of 15 days (*p* = 0.95) indicating that BioXmark^®^ had no significant impact on tumour response. In contrast, the fractionated treatment groups of 2 × 8 Gy and 4 × 4 Gy showed different tumour growth delay characteristics, with the BioXmark^®^-injected tumours showing reduced tumour growth delay compared to control irradiated tumours (Figure 4B).

**Figure 5:**
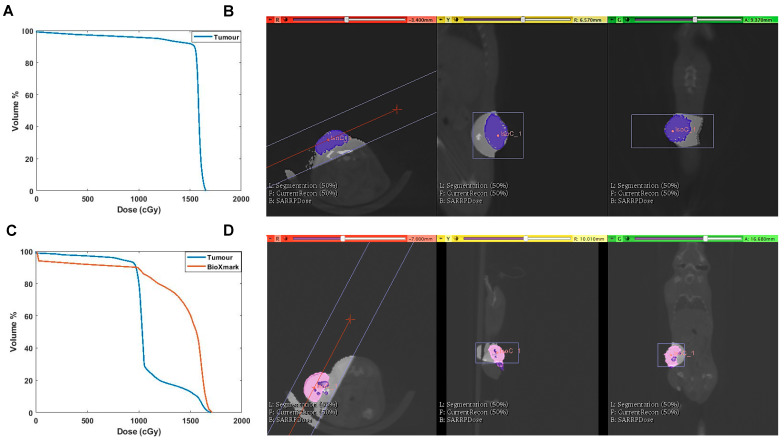
Evidence of underdosing of LLC tumour due to intratumoral injection of BioXmark^®^. (**A**) Dose volume histograms (DVHs) of tumours receiving 16 Gy single-dose irradiation. (**B**) Corresponding treatment plan from Muriplan of CBCT scan of mouse with tumour tissue contoured in blue. (**C**) DVH outlining dose received by tumour tissue and BioXmark^®^ at 16 Gy single-dose irradiation. (**D**) Corresponding treatment plan from Muriplan showing CBCT scan of mouse, with tumour contoured in pink and intratumoral injection of BioXmark^®^ in purple.

Dose volume histograms (DVH) extracted from the treatment planning software, Muriplan, showed that injection of BioXmark^®^ affected the dose calculations by producing imaging artefacts which reduced the actual dose delivered to the irradiated tumours (Figure 5), with the CBCT values for all materials involved presented in Table S1. Treatment planning software detected the high density of BioXmark^®^, equivalent to bone, which led to the calculation of a lower dose. 

Consequently, tumours containing BioXmark^®^ repeatedly received a lower dose than irradiated control animals and so the fractionated treatment groups had been underdosed; this effect became important with the increase in the number of fractions delivered. The effects were more significant for fractionated doses due to a lower biological effective dose (BED) compared to single-dose exposures (Table 1).

Retrospective analysis was undertaken through replanning the beam isocentres and determining the updated dose calculations for BioXmark^®^ treatment groups. Initial treatment plans used BioXmark^®^ as the isocentre placement for beam orientation, whereas for the second round of planning, this was used for guidance only, with the isocentre placed adjacent to the injected BioXmark^®^. Movement of the isocentre millimetres away from the injected BioXmark^®^ lead to an even dose distribution to the tumour, and thus the DVHs produced from this replanning resulted in a recalculated dose similar to the dose delivered to the irradiated only animals and the prescribed dose (Figure S2).

## 3. Discussion

In this study, we evaluated the performance of BioXmark^®^ as an injectable liquid fiducial marker for imaging applications on small animal radiotherapy platforms. SAIGRT is the current state-of-the-art technology in experimental radiobiology, and is enabling previously unachievable approaches to closely mimic clinical exposure scenarios in laboratory studies [5,32,33,34]. In these systems, onboard CBCT imaging can potentially deliver volumetric imaging of soft tissues, yet there remain considerable challenges associated with delineation of soft tissues, targeting of small volumes and standardisation of imaging and treatment planning set-ups that vary between laboratories [28,32,35,36].

Solid fiducial markers have clinically proven to be beneficial in treatment targeting and alignment, but have not reverse-translated into preclinical applications due to implantation challenges, large imaging artefacts and the potential for dose perturbations in the target volumes [11,17,19,20,22]. Recently, a radio-opaque solid marker was used for image guidance in a mouse model of radiation-induced intestinal damage [37]. However, this approach required surgical implantation into the jejunum, with associated increased risk of adverse events in experimental mice.

An alternative strategy, more applicable to mouse models, is the use of injectable fiducial markers. BioXmark^®^ is a robust, radio-opaque injectable marker that has been clinically evaluated in multiple indications including oesophageal, breast and lung cancers [25,38,39,40,41]. BioXmark^®^ is composed of biodegradable sucrose acetate isobutyrate (SAIB), an iodine contrast agent in an ethanol solvent which diffuses out of the marker after being injected into soft tissue, increasing viscosity to form a radio-opaque, semi-solid, gel-like marker [42]. In comparison to solid fiducial markers, this formulation can be injected at various volumes specific to individual patients [23,43]. A previous study demonstrated the benefit of BioXmark^®^ in an orthotopic pancreatic tumour model for improving soft tissue delineation, repositioning and fusion of treatment plans [24]. However, detailed in phantom and in vivo imaging analysis and characterisation of radiobiological response after the injection of BioXmark^®^ are yet to be reported.

In the current study, BioXmark^®^ was shown to produce significantly reduced CBCT imaging artefacts compared to solid gold- and polymer-based markers. Imaging artefacts produced by solid markers negatively impact the visualisation of small structures which are important during SAIGRT. However, volumes as small as 10 μL of BioXmark^®^ could equally enhance CBCT image contrast without producing disruptive imaging artefacts and clearly enabled visualisation of anatomical structures in small animals. In comparison to solid fiducial markers, the observed reduction in CBCT artefacts for BioXmark^®^ may also improve segmentation and uncertainty during dose calculation. From our data, volumes of BioXmark^®^ >60 μL caused imaging artefacts comparable to solid markers and are unsuitable for SAIGRT.

In addition to image quality, several aspects need to be considered in the preclinical setting, including stability, in vivo contrast enhancement and potential impacts on dose calculation. BioXmark^®^ was shown to be safe and stable in vivo when administered via subcutaneous and intratumoral injection routes. It was also shown to be stable with minimal migration from the injection site for time periods up to 5 months, again supporting its use in preclinical radiotherapy studies. These findings are in agreement with previous reports of high tolerability and stability in mice up to 4 months after injection [24].

Our data showed that BioXmark^®^ may impact dose calculations when planning to use an isocentre located within the injected fiducial volume. This caused BioXmark^®^ to be recognised as a high-Z material during dose calculation, resulting in observable differences in tumour growth delay. These effects may be significant during radiobiological studies and could be minimised by removing BioXmark^®^ from the point-dose calculation. This could be achieved by injecting BioXmark^®^ into an adjacent peri-tumoral site or by placing the isocentre outside of the injected volume. In addition, the observed limitations of BioXmark^®^ highlight the need for robust imaging quality assurance that can be achieved through the use of standardised imaging phantoms. Furthermore, advanced image reconstruction techniques and imaging methods such as dual-energy CT (DECT) may also reduce uncertainties [44]. Ultimately, Monte-Carlo-based treatment planning calculations may be more appropriate when using BioXmark^®^, offering improved ability to segment multiple tissues based on assumed atomic numbers calculated from CBCT images [45,46].

In this study, we presented the first data evaluating the effects of BioXmark^®^ on tumour response in vivo from both single and fractionated exposures. BioXmark^®^ was shown to have no significant impact on tumour growth in control animals. In irradiated animals, there was an observable but not statistically significant difference in tumour growth delay, which was related to the number of fractions delivered. We hypothesised that these variations were due to the high mass attenuation coefficient assigned to the BioXmark^®^ during dose calculation, resulting in underdosing of the adjacent tumour tissue. Further investigation of the DVHs and contouring of BioXmark^®^ in Muriplan confirmed these findings and replanning of treatments lead to more accurate dose calculations.

## 4. Materials and Methods

### 4.1. Liquid Fiducial Marker

The novel liquid fiducial marker used in this study was BioXmark^®^, produced by Nanovi A/S (Kongens Lyngby, Denmark). BioXmark^®^ is a sterile, ready-to-inject fiducial maker composed of biodegradable sucrose acetate isobutyrate (SAIB), iodinated SAIB and ethanol as a solvent. This formulation ensures that when BioXmark^®^ is injected into soft tissue, the ethanol partly diffuses out of the marker, increasing the viscosity of the marker and resulting in the formation of a semi-solid gel [42]. Aspiration from the ampoule was completed using an 18 gauge needle and injection into in phantom and in vivo models was done with a 25 gauge needle.

### 4.2. Solid Fiducial Markers

Commercially available gold (Gold Anchor^TM^, 0.4 mm in diameter and 3 mm in length) and polymer (PolyMark^TM^, 1 mm in diameter and 3 mm in length) fiducial markers were used to compare in phantom imaging artefacts on CBCT imaging with BioXmark^®^.

### 4.3. Imaging and Irradiation

Mice were irradiated with 220 kVp X-rays under CBCT image guidance using a Small Animal Radiation Research Platform (SARRP, Xstrahl Life Sciences, Camberley, UK) calibrated using the Institute of Physics and Engineering in Medicine and Biology (IPEMB) code of practice [47], using ionizing chambers and gafchromic film. The half-value layer (HVL) was 0.65 mm Cu with a 0.15 mm Cu filter, for a 220 kV treatment beam with the dose rate quoted being the surface dose rate.

Mice were randomized prior to irradiation, with a single fraction dose of 16 Gy or fractionated doses of 2 × 8 Gy or 4 × 4 Gy delivered using a parallel opposed, anterior-posterior beam geometry, with equally weighted doses, with a 10 × 10 mm collimator (dose rate 2.67 ± 0.11 Gy/min for 34 cm SSD). CBCT scans were performed before irradiation and DVHs calculated for each mouse using Muriplan (Xstrahl Inc, Suwannee, GA, USA). Muriplan uses a heterogeneous superposition convolution dose engine implemented on a graphics processing unit (GPU), and the quoted dose is the dose to medium. The entire small animal treatment was controlled from the 3DSlicer-based user interface. The acquired CBCT was transformed into material properties by defining five discrete windows for air, lung, fat, tissue and bone (Table S2). The dose engine uses the absorption coefficients of these materials according to the National Institute of Standards and Technology database [48].

### 4.4. In Phantom Studies

A 3D-printed phantom (4 × 4 × 4 cm) filled with gelatine from porcine skin (50 mg/mL, Sigma-Aldrich Company, LTD, Dorset, UK) was used to replicate soft tissue. A layer of gelatine (1 cm) was added to the phantom before and after the placement of each marker. The gold and polymer fiducial markers were placed into the gelatine using tweezers and 20 μL of BioXmark^®^ was injected using a 25 gauge needle. Gelatin was set in the fridge for 1 h and then imaged using CBCT. Phantom models were imaged twice at 40, 50 and 60 kV.

#### Artefact Analysis

CBCT scan slices were individually analysed using ImageJ software (http://imagej.nih.gov/ij). The surrounding artefact area was determined by adjusting the threshold of images to highlight imaging artefacts (black) from the background (white), and then utilising the ‘analyse particles’ function to calculate the relative artefact area. Artefact area was measured in pixels (px), (1 px = 0.26 mm). For each marker, CBCT scan slices covering each marker were combined and averaged. Scans were completed twice at 40, 50 and 60 kV and results averaged for each.

### 4.5. In Vivo Studies

Tumour growth and stability were investigated in female 12–15-week old C57BL/6J mice obtained from Charles River Laboratories (Oxford, UK). All mice were housed under controlled conditions (12 h light–dark cycle, 21 °C) in standard caging with three to five littermates, and received a standard laboratory diet and water ad libitum. To improve the welfare of mice, environmental enrichment tools were placed in caging such as cardboard tubes for exploration, softwood blocks to encourage gnawing to prevent tooth overgrowth, nesting material for comfort and mouse swings for added cage complexity and exercise. Mice were also handled gently and frequently from a young age to reduce stress.

Prior to irradiation, animals were anaesthetised with ketamine and xylazine (100 mg/kg and 10 mg/kg) by intraperitoneal injection. All experimental procedures were carried out in accordance with the Home Office Guidance on the Operation of the Animals (Scientific Procedures) Act 1986, published by Her Majesty’s Stationary Office, London, and approved by the Queen’s University Belfast Animal Welfare and Ethical Review Body (PPL2813). Animal studies are reported in compliance with the ARRIVE guidelines [49].

#### 4.5.1. In Vivo Stability

Mice were administered anaesthesia via intraperitoneal injection. Different volumes of BioXmark^®^ (10, 20 or 40 μL) were injected at two separate points on the flank (right and left) or one intraperitoneal injection (40 μL) using a 25 G needle (*n* = 4). Mice were then imaged on CBCT at various time points over a 5-month period. The weight and wellbeing of the mice was assessed weekly. The CBCT value, migration and volume of BioXmark^®^ visible on CBCT scans was assessed throughout the study. Analysis was performed using Muriplan treatment planning software.

#### 4.5.2. In Vivo Tumour Model

Tumour xenograft studies were performed using Lewis lung carcinoma (LLC) cells cultured in DMEM media (+4.5 g/L D-glucose, L-glutamine, -pyruvate) supplemented with 10% foetal bovine serum (FBS), 1% penicillin/streptomycin and 1 μM sodium pyruvate. Cells were maintained at 37 °C in a humidified atmosphere of 5% CO_2_ and subcultured every 3–4 days to maintain exponential growth.

LLC cells were cultured in vitro and prepared in PBS (1 × 10^6^ cells per 100 μL). Subsequently, 100 μL was injected subcutaneously into the flank of each C57BL/6 mouse (*n* = 48). Mice were anesthetized using inhalant isoflurane for implant and placed in a heat box for recovery. Mice were then returned to conventional housing and closely monitored.

Tumour volume was determined three times a week using calliper measurements in three orthogonal dimensions. After tumour growth to a volume of 100 mm^3^, mice were randomly allocated to one of the following subgroups: (i) control, (ii) control + BioXmark^®^, (iii) single dose: 16 Gy, (iv) single dose: 16 Gy + BioXmark^®^, (v) fractionated dose: 2 × 8 Gy, (vi) fractionated dose: 2 × 8 Gy + BioXmark^®^, (vii) fractionated dose: 4 × 4 Gy, (viii) fractionated dose: 4 × 4 Gy + BioXmark^®^. An average of six mice were allocated to each subgroup through randomisation. Mice were ear-punched for identification. Intratumoral injection of 20 μL of BioXmark^®^ was performed immediately prior to irradiation, accounting for approximately 20% of the pre-treatment tumour volume. Experimental endpoints were defined as tumour volume exceeding 500 mm^3^ (<GMD 12 mm) or loss of 15% body weight.

### 4.6. Statistical Analysis

Statistical differences between populations were calculated using unpaired two-tailed Student’s *t*-tests, or one-way ANOVA tests where appropriate, with a significance threshold of *p* < 0.05 using Prism GraphPad Prism 7 (Version 7.01, GraphPad Software, Inc., San Diego, CA, USA) (www.graphpad.com). Data are presented as the average for the entire experimental arm ± SEM.

## 5. Conclusions

Our data further support the use of BioXmark^®^ in preclinical imaging and SAIGRT applications, based on reduced imaging artefacts with minimal impacts on tumour response. Alterations in CT number due to BioXmark^®^ should be carefully considered during dose calculations to ensure homogeneity. BioXmark^®^ is a useful tool for improving target definition and alignment protocols in SAIGRT studies within the NC3Rs framework.

## Figures and Tables

**Table 1:** Summary of treatment parameters following single-fraction and fractionated radiotherapy exposures in combination with BioXmark^®^. Base on the I in vitro dose response of LLC cells, an α/β ratio of 3.1 was used to calculate the BED and EQD_2_. Treatment plans (Muriplan software, Xstrahl Inc, Suwannee, GA, USA) were used to calculate the total dose delivered; an average of six mice were allocated to each subgroup and results were averaged.

Dose Schedule	BED (Gy)	EQD_2_ (Gy)	Average Total Dose Delivered (cGy ± SEM)	Difference in Dose Compared to Non-BioXmark^®^ Control (%)
4 × 4 Gy	36.6	22.3	1609 ± 7	
4 × 4 Gy + BioXmark^®^			1442 ± 113	−10.4
2 × 8 Gy	57.3	34.8	1606 ± 8	
2 × 8 Gy + BioXmark^®^			1254 ± 153	−21.9
16 Gy	98.6	59.9	1611 ± 14	
16 Gy + BioXmark^®^			1307 ± 160	−18.8

## Supplementary Materials

The following are available online at https://www.mdpi.com/2072-6694/12/5/1276/s1. Figure S1: In vivo stability of BioXmark^®^ after intraperitoneal injection, Figure S2: Re-planned Dose Volume Histogram (DVH) of an LLC tumour receiving single dose 16 Gy irradiation with an intra-tumoral injection of BioXmark^®^, Table S1: Average CBCT values for air, tissue, bone and BioXmark for control and BioXmark implanted mice, Table S2: Maximum values for segmentation windows for control and BioXmark implanted mice.
