# Supplementary material for: Evaluation of a Novel Liquid Fiducial Marker, BioXmark®, for Small Animal Image-Guided Radiotherapy Applications
Source: Cancers (Basel). 2020 May 18;12(5):1276. doi: 10.3390/cancers12051276 (PMC7280978; doi:10.3390/cancers12051276)
Supplement: Supplementary file 1 [file cancers-12-01276-s001.pdf]

Supplementary Materials

Evaluation of a Novel Liquid Fiducial Marker, BioXmark®, for Small Animal Image-Guided Radiotherapy Applications

Kathryn H. Brown, Mihaela Ghita, Giuseppe Schettino, Kevin M. Prise and Karl T. Butterworth

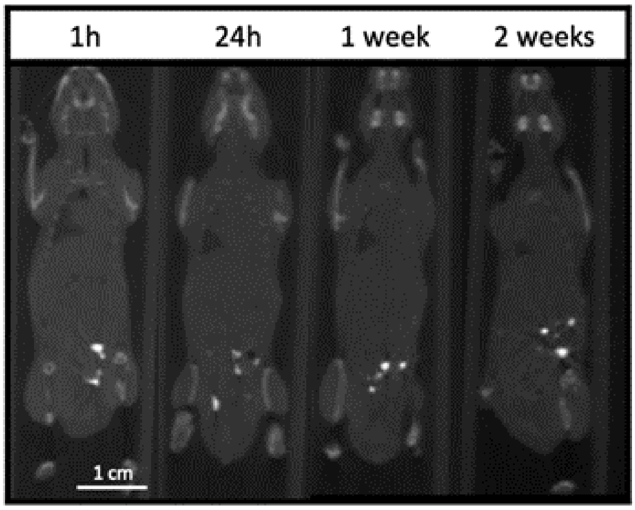

Figure S1. In vivo stability of BioXmark® after intraperitoneal injection. Coronal CBCT scans at 1 hour, 24 hours, 1 week and 2 weeks post intraperitoneal injection of 40 μL of BioXmark®.

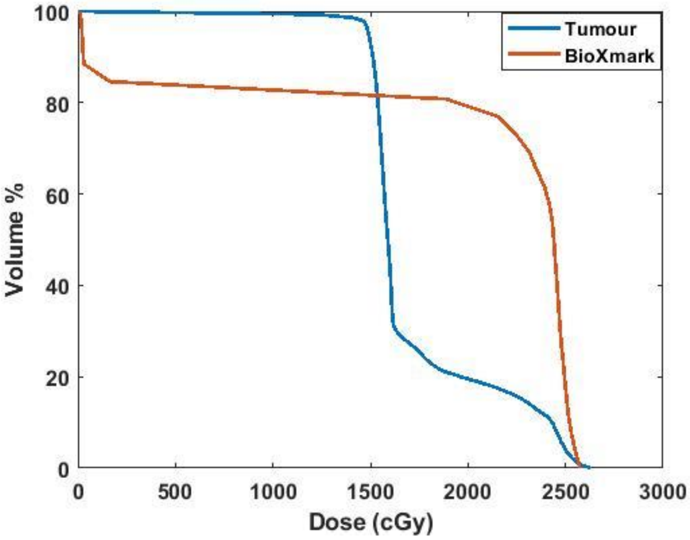

Figure S2. Re-planned Dose Volume Histogram (DVH) of an LLC tumour receiving single dose 16 Gy irradiation with an intra-tumoral injection of BioXmark®. Retrospective analysis was completed, on Muriplan software, by re-planning the beam iso-centre millimetres away from intra-tumoral BioXmark® and a new DVHs determined. This DVH outlines the dose that would be received by BioXmark® and tumour tissue after re-planning.

Table S1. Average CBCT values for air, tissue, bone and BioXmark for control and BioXmark implanted mice.

Material	Control		BioXmark	
	Average CBCT	SEM	Average CBCT	SEM
**Air**	15345.6	76.91	10848.75	125.74
**Tissue**	18897.6	126.86	13572.25	395.73
**Bone**	19402.4	215.02	17104.75	195.47
**BioXmark**			22915.25	1325.36

Table S2. Maximum values for segmentation windows for control and BioXmark implanted mice.

Material	Segmentation Control	Segmentation BioXmark
Air	16494	12000
Lung	17000	12500
Fat	17502	13000
Tissue	20500	15000
Bone	65535	65535

© 2020 by the authors. Licensee MDPI, Basel, Switzerland. This article is an open access article distributed under the terms and conditions of the Creative Commons Attribution (CC BY) license (http://creativecommons.org/licenses/by/4.0/).
